# Simulation of Linear and Cyclic Alkanes with Second-Order Møller–Plesset Perturbation Theory through Adaptive Force Matching

**DOI:** 10.1021/acs.jctc.4c00509

**Published:** 2024-06-07

**Authors:** Alexei Nikitin, Feng Wang

**Affiliations:** Department of Chemistry and Biochemistry, University of Arkansas, Fayetteville, Arkansas 72701, United States

## Abstract

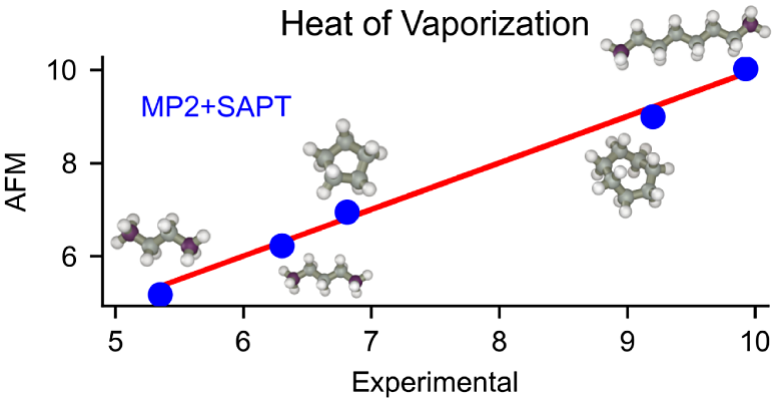

Predicting ensemble properties, such as density and heat of vaporization, of small hydrocarbons is challenging due to the dispersion-dominated weak interactions between these molecules. With the adaptive force matching (AFM) method, the bonded and short-range nonbonded interactions are fitted to second-order Møller–Plesset perturbation theory (MP2) references computed with the def2-TZVP basis set. The dispersion is modeled using symmetry adapted perturbation theory (SAPT) at MP4 accuracy using the def2-TZVPD basis set. A new charge matrix decomposition technique is described to obtain partial charges in AFM. Although the models developed do not have any empirical parameters, several properties of the resulting models are compared with experiments as validations. The density, heat of vaporization, pressure dependence of density, diffusion constants, and surface tensions all show quantitative agreement with experiments. Although the density shows a very small systematic error, which could be due to missing three-body dispersion, the heat of vaporization agrees with experiments of within 0.5%. The paper shows that AFM can be used as a reliable tool to enable simulations at post-Hartree–Fock quality at the cost of molecular mechanics force fields.

## Introduction

*Ab initio* molecular dynamics (AIMD) at the post-Hartree–Fock level is associated with tremendous computational cost. This practical challenge leads to the use of density functional theory (DFT) for most AIMD simulations despite the fact that DFT is known to suffer from various limitations,^1−5^ such as the charge-delocalization error and problems with modeling dispersion. Nonpolar organic molecules, such as hydrocarbons, are especially challenging for DFT as the intermolecular interaction is dominated by dispersion.

Hydrocarbons are an important class of molecules, as they are the basis of organic chemistry and the main component of crude oil. Many small-molecule hydrocarbons are being studied as greener alternatives to chlorofluorocarbon as refrigerants due to their lower environmental impact. Hydrocarbons are also quite abundant in the outer solar system; for example, the lakes of Titan, the largest moon of Saturn, are filled with hydrocarbons.^6^ Highly accurate simulations of hydrocarbons thus help to answer questions in various fields spanning organic, petroleum, environmental, and planetary chemistry.

Since the early days of molecular mechanics simulations, force fields for hydrocarbons have been in development.^7−9^ They have been continuously refined over the years as they are an important part of lipids and are common components of drug molecules.^10−13^ Early force fields described hydrocarbons of small molecular sizes well but gave poor results for extended linear alkanes.^14^ It is important to point out that the vast majority of empirical models for hydrocarbons uses the Lennard-Jones potential for short-range repulsion and long-range dispersion. The 1/*r*^12^ repulsion in the Lennard-Jones potential is not very physical, as the true short-range repulsion is mostly a result of exchange repulsion, where the antisymmetric Fermion nature of electrons keeps them from occupying the same spatial orbital with the same spin.^15^ The exchange repulsion is better approximated using an exponential term rather than a power law.^16,17^

In this study, the possibility of investigating the properties of hydrocarbons by force matching the second-order Møller–Plesset perturbation theory (MP2)^18,19^ with the adaptive force matching (AFM) method^20^ will be explored. Direct MP2 molecular dynamics simulations will not be practical for systems with greater than a few dozen molecules. AFM maps the MP2 potential energy surfaces (PES) onto physics-based energy expressions, which are, in turn, used to model various hydrocarbons. We will study ensemble properties that can be directly confirmed with experiments as a validation of both the AFM method and the MP2 reference. However, none of the properties will be fit during the development of the models.

Although AFM fits energy expressions similar to those used in traditional force fields, unlike a traditional force field, AFM uses pair-specific parameters rather than atomic parameters for repulsion and dispersion. In other words, no combining rules are used to derive interactions between different types of atoms. The only atomistic parameters used by AFM models are partial charges. Also, AFM models are fit with the sole objective of reproducing the atomic and molecular forces computed with the reference electronic structure method, with no adjustments to fit experiments. Thus, AFM is better understood as a proxy to perform electronic structure simulations rather than a traditional force field development protocol.

AFM has shown good success for predicting properties of small electrolytes^21−23^ and nonelectrolytes^24−26^ in water based on either MP2 or DFT reference forces. One challenge in creating hydrocarbon models is the small heat of vaporization of such molecules of slightly more than 1 kcal/mol per carbon, which is not much more than the thermal energy of 0.6 kcal/mol at room temperature. Thus, any minor imperfection in the reference method or the fitting procedure could lead to models with large percentage errors.

Being nonpolar weakly interacting molecules, dispersion is a dominant component of the cohesive energy. Most DFT-based simulations supplement the DFT energy with an empirical dispersion correction.^27,28^ The ability of AFM to fit to the post-HF method avoids the need to rely on an empirical description of dispersion, thus allowing for a more rigorous description of dispersion for hydrocarbons.

In this work, MP2-based models will be developed for *n*-butane, *n*-pentane, *n*-octane, cyclopentane, and cycloheptane with AFM. *n*-Butane, cyclopentane, and *n*-pentane are especially challenging since their boiling temperatures are below or close to ambient temperature. The low boiling temperature is correlated with the heat of vaporization as
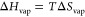
1where Δ*S*_vap_ is approximately 85 J/(mol·K) according to Trouton’s rule. A minor inaccuracy in the Δ*H*_vap_ could lead to too low of a boiling temperature and spontaneous vaporization under the simulation condition. The two cycloalkanes were chosen due to the drastic difference in strain. While cyclopentane does not exhibit much strain, cycloheptane does not have unstrained conformations. The *n*-octane is chosen to see if the AFM mapped models would suffer from problems for extended linear hydrocarbons.^12^

## Methodology

### Adaptive Force Matching

The first example of force matching-based potential development is probably by Ercolessi and Adams.^29^ The Ercolessi and Adams work fitted DFT forces and constrained parameters to reproduce experiments. In 2004, Izvekov et al.^30^ fitted a water model with force matching relying on DFT forces obtained from a Car–Parrinello molecular dynamics (CPMD) trajectory. However, the fit by Izvekov used cubic splines rather than a physics-based energy expression. Many groups have attempted force matching,^31−34^ with some of the recent work combining force matching with machine learning.^35^

Compared to other force matching techniques, one unique strength of AFM is that it allows the use of post-Hartree–Fock methods with quantum mechanics/molecular mechanics (QM/MM)-based model training. AFM has showed great success in fitting various aqueous systems and some simple nonaqueous system, such as CO_2_^36^ and graphene.^37^

The detailed steps of AFM have been described in the past.^20,26,38^ Briefly, AFM has three steps: the sampling step, which typically employs molecular dynamics (MD) to produce a set of conformations to use as the training set; the QM/MM step, which is used to compute QM reference forces for the training set; and the FM step, which is used to fit the force field. The force field from the FM step then starts another iteration of AFM, where both the sampling and the QM/MM steps can benefit from the improved MM model from the previous generation.

The detailed procedure for using AFM to create the alkane models will be provided as Supporting Information, with only some key information summarized here. In addition, we will describe a charge matrix decomposition scheme, which is needed to obtain partial charges for molecules, such as *n*-butane, *n*-pentane, and *n*-octane.

**Figure 1:**
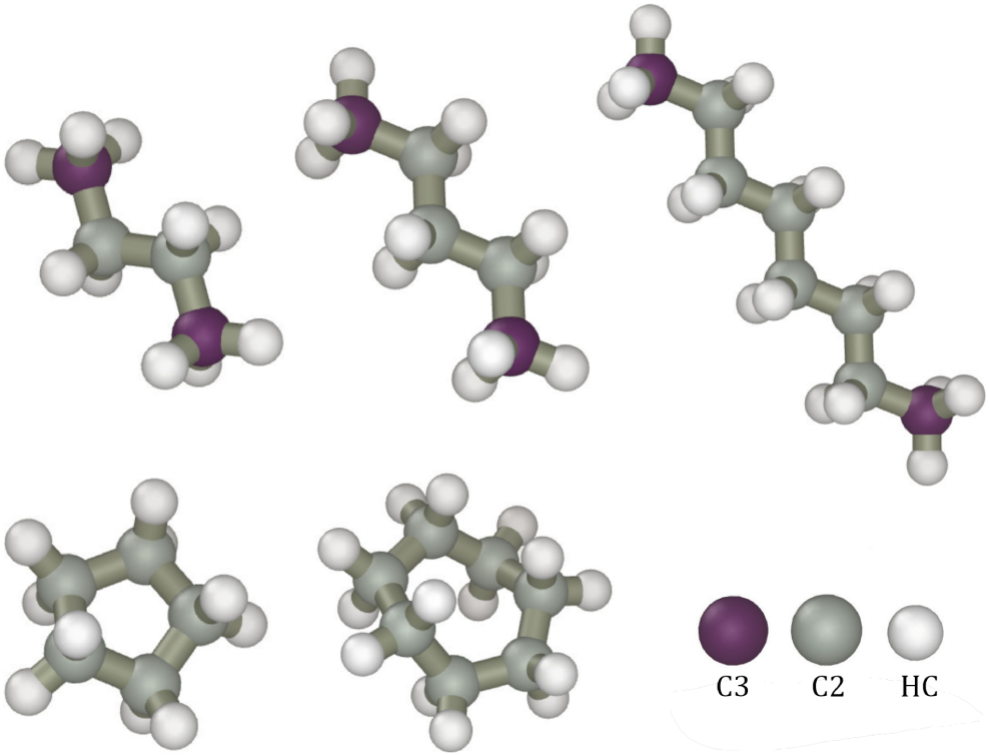
Atom types for the molecules were studied. Note that the same types of atoms in different molecules do not use the same parameters unless otherwise discussed.

AFM generally treats each symmetry unique atom as different. While this leads to two atom types for cyclic alkanes, more are needed for normal alkanes. To simplify the model and improve numerical stability, three atom types are used for the three normal alkanes as shown in Figure 1. In our procedure, all secondary carbons are treated as equivalent and assigned the type C2, and all the H atoms use the same type HC. While this is a good approximation for pure alkanes, such simple atom typing probably will not be sufficient for substituted hydrocarbons, where atoms of drastically different electronegativities are present.

For hydrocarbons, the dispersion is of paramount importance. In AFM, dispersion is generally fit to SAPT^15^ or Grimme’s empirical dispersion correction to DFT^27^ before the AFM iterations. In this work, we will fit to E^2^ dispersion^39^ from SAPT calculations. It is worth mentioning that being a double perturbation theory, the SAPT E^2^ corresponds to dispersion at the MP4 level of theory. The dispersion in standard MP2 couples only the excitations of Hartree–Fock orbitals.

For each molecule, the SAPT E^2^ dispersion will be computed with the Psi4 program^40^ using def2-TZVPD^41^ along with a dimer-centered basis set approach. Alkane dimers are extracted from liquid phase simulations, with the nearest atom distance ranging from 5 to 12 Å. This range is chosen since exchange-dispersion, which is not being considered in our fitting, may not be negligible at shorter distances. Fitting only to dimers with larger separations will ensure that the dispersion-related terms in the AFM model pick up the correct asymptotic behavior. Due to the important role of dispersion for these weakly bound molecules, we include both C_6_ and C_8_ terms. Our tests including an even higher-order C_10_ term led to an instable model, where the C_10_ term was fitted to be repulsive.

Since Psi4 SAPT calculations only provide energies rather than forces, the number of equations to fit dispersion is generally far less than the number of equations used in a typical FM step of AFM. To reduce statistical noise and improve numerical stability, it was decided to use the same dispersion parameters between the same types of atoms regardless of the molecule type. This is accomplished by fitting all the dispersion parameters together, combining reference E^2^ energies from SAPT calculations on homodimers of *n*-butane, *n*-pentane, and *n*-octane. Such dispersion parameters are also used for the two cycloalkanes. After the dispersion parameters are determined, the AFM iterations are performed with the empirical X75 force field^42^ as the initial guess model. The sampling step includes two separate MD simulations at 298 and 328 K with 100 conformations obtained from each temperature to be used as the training set. Instead of obtaining the reference forces using QM/MM, we decided to perform QM cluster calculations with 6 alkane molecules modeled with QM without a MM region. Since hydrocarbons are nonpolar, there is not much benefit in keeping the MM particles for electrostatic embedding. Omitting MM particles will eliminate the QM/MM boundary. AFM generally eschews using any forces near the QM/MM boundary to avoid artifacts. Thus, eliminating MM particles will allow AFM to use forces on all QM particles. The QM reference method is RI-MP2^43^ using RIJCOSX approximation^44^ and the def2-TZVP basis set.^41^

AFM is an iterative procedure. An effective method to judge convergence is to examine the radial distribution functions (RDFs) of each generation and consider the generations converged when such RDFs stop changing. With the X75 initial guess, convergence is obtained quickly in one or two generations. All parameters of the final force field are obtained by global fitting using conformations from the last 4 generations of AFM.

### Charge Matrix Decomposition (CMD)

Nonlinear optimization is an NP hard problem. To ensure robust optimization, AFM relies on a system of linear equations of the form
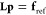
2where **L** is an *N* × *M* matrix. The number of columns, *M*, is the number of parameters; and the number of rows, *N*, is the number of forces. The *N* reference forces form column vector **f**_ref_. The vector **p** is the parameter vector. Typically in AFM, the number of forces is far greater than the number of parameters, and eq 2 can only be solved in a least-squares sense. We currently solve such a problem with singular value decomposition (SVD).^45^

With this formalism, AFM does not directly determine the partial charges of each atom. Instead, it fits charge products as they are linear parameters of the Coulombic forces. The AFM fitting code, CReate Your Own Force Field (CRYOFF) treats all charge products as independent parameters, thus in general the charge products do not satisfy
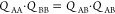
3where *Q* indicates a charge product, with the subscripts being atom types. For example, *Q*_AB_ in eq 3 is the product of charges *q*_A_ and *q*_B_. It can be shown that with proper charge neutrality constraint equations, eq 3 is satisfied when there are only two types of atoms. Thus, when developing models^36,46^ for water and CO_2_, there is no issue in solving for partial charges from the charge products. When fitting solute charges in water,^22^ the atomic charges of the solute are obtained from charge products with water. Although eq 3 is not satisfied for charge products between solute atoms, it will not cause any problem since solute–solute interactions are determined by a second intramolecular fitting step that enforces eq 3 before other intramolecular bonded and nonbonded parameters are determined.

When fitting a liquid alkane with more than 2 atom types, the violation of eq 3 will lead to the inability of obtaining a set of charges that gives the same forces as those obtained by the least-squares solution of eq 2. To solve this problem, we eigendecompose the charge product matrix, which is real and symmetric, to obtain the eigenvalues λ_*i*_ along with the eigenvectors. We then approximate the charge product matrix as
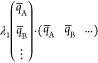
4where λ_1_ is the largest eigenvalue and
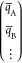
5is the corresponding eigenvector. The actual atomic charge on atom A is
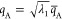
6

The sign of the eigenvector in eq 5 is arbitrary. This is expected as flipping the sign of all atomic charges will not change any charge products and hence will not change the Coulombic forces. For the development of alkane models, we pick the sign so that the hydrogen atom carries a positive charge. In general, it is probably best to define a hydrogen atom bound to the most electronegative element to be positive. In the case that no hydrogen is present, or in the case of metal hydrides, choosing the least electronegative atom to be positive is likely to be appropriate.

We note that this is the same procedure as principal component analysis.^47^ An eigen-decomposition can be considered as a special case of SVD, except that with SVD, the convention is to require the singular values not to be negative. In an eigen decomposition, the eigen value can be any real number. A negative eigenvalue in eq 4 can be interpreted as meaning that the particles have imaginary charge values. Thus, for the purpose of determining the optimal charges, we are only interested in the largest positive eigenvalue. In principle, it is possible that the charge product matrix has no positive eigenvalues. We have never encountered that in practice. We believe that the best decision in such cases would be to assign a charge of zero to all the atoms.

With CMD, the intermolecular parameters of AFM are determined by solving eq 2 twice. For the first time, all of the charge products are treated as independent parameters. CMD is then performed using the charge product matrix to obtain partial charges. The charge products will then be derived based on the partial charges thus obtained (eq 6). The derived charge products will be used to solve eq 2 again to obtain other nonCoulombic intermolecular parameters. With only two atom types, the two cyclic alkanes do not require such a CMD procedure, and all intermolecular parameters are determined by solving eq 2 only once.

### Property Calculations

After the models are developed, property calculations are performed with the Gromacs^48^ 2019.6 package. The models are validated by computing the density of the liquid, the heat of vaporization, the density as a function of pressure, the diffusion constant, and the surface tension. Whenever possible, we will compute the property and compare it with experimental values at 298 K except for *n*-butane, where we computed the properties at the experimental boiling temperature of 272 K. For diffusion constants, experimental values at the desired temperature are not always available. We computed the diffusion constants at the same temperature as the available experimental data. The details for these simulations are reported in Supporting Information.

It is worth mentioning that while the Gromacs package computes the correction to energy and stress due to truncation of long-range van der Waals interactions, the correction considers only the truncation of the 1/*r*^6^ term. In our study, dispersion has both 1/*r*^6^ and 1/*r*^8^ terms, and the magnitude of the coefficient of the 1/*r*^8^ term is fairly large. For example, for the *n*-butane model, the truncation of the 1/*r*^8^ term at 1.0 nm will lead to an overestimation of the pressure by 32.2 bar. The overestimation reduces quickly with larger van der Waals cutoff and becomes 8.7 bar at 1.3 nm and drops to below 1.0 bar at 2.0 nm. The computational efficiency depends sensitively on the cutoff radius. We thus simulate the various hydrocarbons with a 1.3 nm cutoff for most property calculations and apply the missing long-range correction to energy and stress manually. For example, to simulate the property of *n*-butane, the barostat will control the pressure at 9.7 bar. The final system will be at 1 bar, considering that the addition of the long-range tail of the 1/*r*^8^ dispersion beyond the cutoff radius will reduce system pressure by 8.7 bar. Similarly, a small correction to the energy is applied when computing the heat of vaporization, although the correction to the energy is smaller than the uncertainty and is negligible in all practical senses. The formula for the long-range correction to the energy and stress due to the 1/*r*^8^ term is derived and reported in the Supporting Information. For the computation of surface tension, a 2.0 nm cutoff is used as the system is inhomogeneous and the standard formula for long-range correction to energy and stress is inappropriate for such systems.^49^

## Results and Discussion

The energy expressions of the force field models are summarized below. The bonds are described with the harmonic bond terms of the form
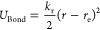
7Both the harmonic constant *k*_r_ and the equilibrium bond length *r*_e_ are being fitted.

The angles are modeled using the harmonic angle term
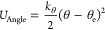
8fitting both *k*_*θ*_ and *θ*_*e*_.

The torsional term is described by using the cosine torsional potential of the form
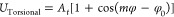
9Since the study only explored saturated hydrocarbon, *m* is set to 3 and φ_0_ is chosen as 0. The coefficient *A*_*t*_ is being fitted. Torsional angles involving hydrogen are not considered.

Noncovalent interactions between atoms *i* and *j* with atom types *a* and *b* are modeled as
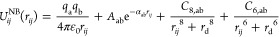
10where *r*_*ij*_ is the distance between atoms *i* and *j* that are separated by more than 2 covalent bonds or in two molecules. The first term on the right-hand side of eq 10 is the electrostatic interaction; the second term is repulsion; the last two terms are short-range-damped dispersion, where *r*_d_ is the damping distance.^50^ The *r*_d_ is chosen based on the van der Waal radius to remove unphysical short-range attraction of the inverse power law and is not fitted during AFM. While the electrostatic and repulsion interactions exist between all pairs of atoms, dispersion terms are placed only between pairs of carbon atoms. The parameters for all of the models are shown in the Supporting Information.

**Figure 2:**
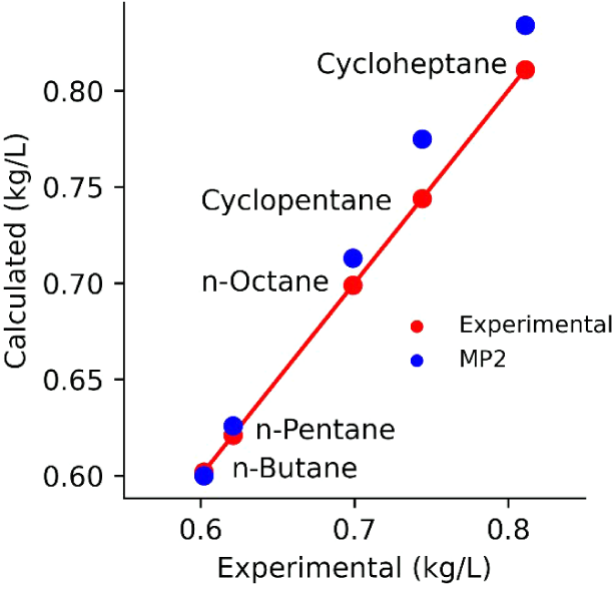
Scatter plot of density obtained using the MP2-based AFM model vs the corresponding experimental values. The diagonal line and the red dots indicate perfect agreement and are used only as guides for the eye.

The densities of the alkanes at 1 bar measured over 20 ns of MD are reported in Table 1 and shown as a scatter plot in Figure 2. The densities of normal alkanes increase as a function of the chain length. Cycloalkanes have a higher density than the corresponding linear alkanes. The cyclopentane density is roughly 20% higher than that of the corresponding *n*-pentane. The cycloheptane density is 25% higher than that of *n*-octane, which is expected to have a higher density than *n*-heptane. The AFM models closely reproduce the experimental densities, giving an error of 0.5% for shorter linear alkanes but around 2% for longer ones. The error for cycloalkanes is slightly higher, reaching 4% for cyclopentane, which is still excellent considering that no experimental data were fit.

**Table 1:** Density (ρ) and Heat of Vaporization for the Alkanes Studied

		ρ (g/L)		Δ*H*_vap_ (kcal/mol)	
substance	*T* (K)	exptl	AFM	exptl	AFM
cyclopentane	298	744^58^	775	6.81^59^	6.951
cycloheptane	298	811^58^	834	9.20^60^	9.000
*n*-butane	272	602^60^	600	5.35^60^	5.178
*n*-pentane	298	621^60^	626	6.3^60^	6.224
*n*-octane	298	699^60^	713	9.926^61^	10.03

It is worth noting that the linearity of the AFM densities is very good, with a Pearson *R*^2^ of 0.999. The slope in Figure 2 is slightly greater than 1, indicating a small systematic error proportional to the density rather than the number of carbons in each molecule. The fitting process ignored 3-body Axilrod–Teller–Muto (ATM) dispersion,^28,51,52^ which is expected to be repulsive and thus led to reduced density. It is highly possible that the correction to pressure due to ATM dispersion grows as the density increases, thus explaining the small systematic error. Although it is possible to compute 3 body dispersion using SAPT^53^ and fit with an Axilrod–Teller–Muto expression or, alternatively, fit to an effective 2 body expression,^36^ we anticipate a large number of configurations to be needed to obtain reasonable numerical stability. Considering that SAPT is a fairly costly method, we postpone such a fit to future investigations.

It is worth reiterating that although the model was fit to reproduce MP2 forces, the SAPT-based dispersion is accurate to the MP4 level. Dispersion is the dominant source of cohesive energy for these alkanes, and having MP4 quality dispersion might explain the high accuracy of the AFM-based models.

The Δ*H*_vap_ was computed with the equation
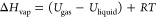
11where *U*_gas_ and *U*_liquid_ are the average potential energies in the gas and liquid phases, respectively.

The average potential energy of the liquid was computed using 30 ns of MD under 1 bar. The average energy of the gas was computed using a single molecule with 100 ns of MD performed without Ewald summation and with the Langevin integrator^54^ and an inverse friction constant of 2.0 ps. Long-range correction to dispersion was calculated for both the *r*_*ij*_^–6^ and *r*_*ij*_^–8^ terms. The latter has to be added manually to *U*_liquid_ as discussed previously.

**Figure 3:**
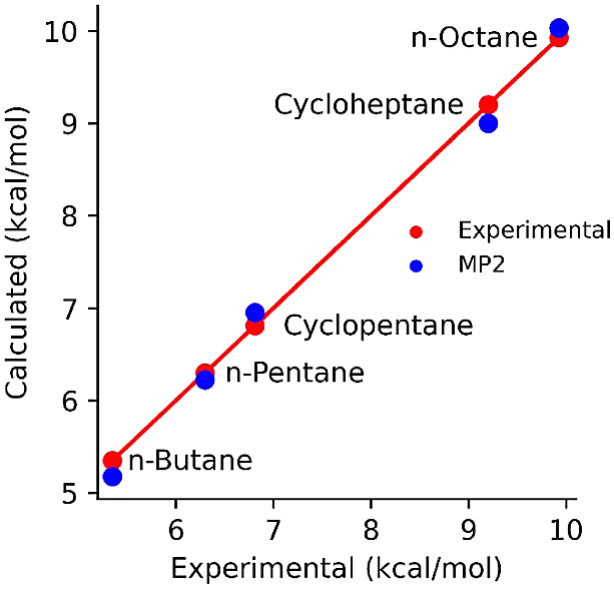
Scatter plot of heat of vaporization obtained using the MP2-based AFM model vs the corresponding experimental values. The diagonal line and the red dots indicate perfect agreement and are used only as guides for the eye.

The Δ*H*_vap_ values of the five alkanes are reported in Table 1 and shown in Figure 3. It is well-known that Δ*H*_vap_ of alkanes is roughly proportional to the number of carbon atoms. The ratio is 1.34 kcal/mol for *n*-butane and roughly 1.25 for the two longer linear alkanes. The per carbon contribution to Δ*H*_vap_ is slightly higher for the cycloalkanes, being 1.34 for cyclopentane and 1.31 for cycloheptane. Note that Δ*H*_vap_ is the energy needed for the molecules to evaporate; the higher strain energy of cycloheptane would affect both the liquid and the gas phases to roughly the same extent and thus would not affect Δ*H*_vap_ much.

The AFM models did an outstanding job of reproducing experimental Δ*H*_vap_. The model Δ*H*_vap_ agrees with experiments within 0.2 kcal/mol for all substances, which is far better than what is generally accepted as the chemical accuracy. We note that this should not be interpreted as nuclear quantum effects (NQEs) being unimportant for alkanes. The C–H vibrations are expected to be quantized. However, for weakly interacting molecules, the intramolecular and intermolecular potential energy surfaces are expected to be largely uncoupled. Thus, the dominant intramolecular component of NQE is expected to be similar in the liquid and gas phases, thus not affecting the prediction of Δ*H*_vap_.

While the AFM models slightly overestimate density, there is no clear indication of systematic error with the Δ*H*_vap_. We note that 3-body dispersion has both intermolecular and intramolecular contributions. While both the intramolecular and intermolecular components of the 3-body dispersion may affect the density, only the missing intermolecular component of the ATM dispersion is expected to affect Δ*H*_vap_.

The diffusion constants *D* of the alkanes are reported in Table 2. The diffusion constants were computed with cubic boxes containing 500 molecules for each alkane. This results in a fairly large box to minimize finite size effects.^55^ It is worth pointing out that the experimental diffusion constants have a fairly large uncertainty. The typical pulsed field gradient NMR performs exponential fitting of the diffusional attenuation, which has uncertainty in both the exact pulse widths used and the range of gradient amplitudes chosen. While a dominant source of error is the spatial nonuniformity of the pulsed field gradients, other important factors that can affect accuracy include sample temperature calibration.^56^ It is thus not surprising that the experimental *D* measured by different groups could differ by 0.2 to 0.3 × 10^–5^ cm^2^/s, which amounts to 15% for cycloheptane and 5% for *n*-pentane. The *D* predicted by AFM models agrees with experiments within 0.3 × 10^–5^ cm^2^/s for all the molecules. For cycloheptane, *n*-pentane, and *n*-octane, the agreement between the model prediction and the experiments is smaller than the variation among different experiments. The largest difference between experiment and simulation is observed for *n*-butane, where there is only one experimental *D* available. This experimental *D* is measured at 190 K. At such a low temperature, the NQE could be more serious. It is possible that NQE will make the cross-section of the *n*-butane larger, making it harder to diffuse than a classical *n*-butane, which would explain the slight overestimation.

**Table 2:** Self-Diffusion Constants of the MP2-Based AFM Models and the Corresponding Experimental Values

		*D* (10^–5^ cm^2^/s)	
substance	*T* (K)	exptl	MP2/AFM
cyclopentane	298	3.09,^62^ 3.1^56^	2.87
cycloheptane	298	1.011,^63^ 0.87^64^	0.90
*n*-butane	195	1.8^65^	2.15
*n*-pentane	298	5.62,^66^ 5.72,^67^ 5.96^68^	5.85
*n*-octane	298	2.356,^63^ 2.45,^69^ 2.55^68^	2.53

**Figure 4:**
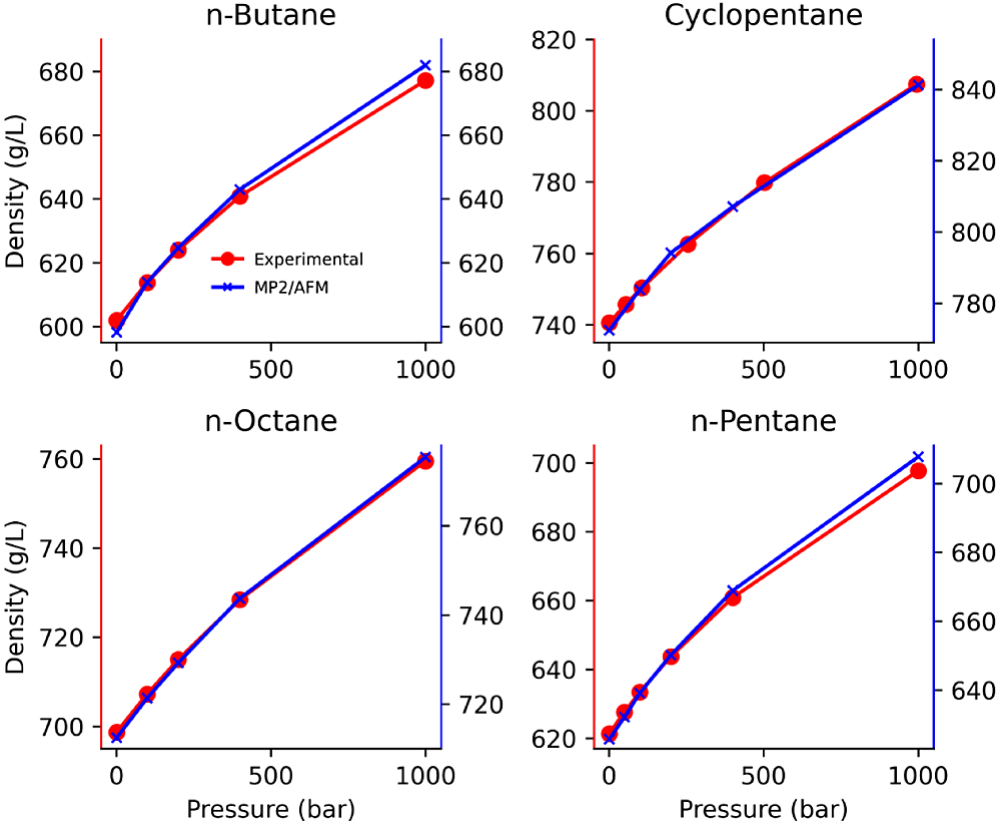
Experimental data for *n*-butane, *n*-pentane, and *n*-octane are taken from ref. (60). Experimental data for cyclopentane is taken from ref. (71).

Figure 4 reports the density as a function of pressure for cyclopentane, *n*-butane, *n*-pentane, and *n*-octane at 298 K except for *n*-butane, which is at 272 K. The density dependence is not shown for cycloheptane due to our inability to find corresponding experimental data in the literature. Since the goal of this figure is to study the pressure dependence, the figure intentionally superimposes the experimental and computed densities at 300 bar by plotting the computed density based on MP2 on the alternative axis. The small error in density at the same pressure shown in Figure 1 is, thus, not visible in this graph. It is clear that MP2-based AFM models faithfully follow the pressure dependence of density up to 1000 bar. We note that the slope of this curve is proportional to the isothermal compressibility:
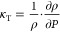
12For cycloheptane, although we were unable to find experimental density as a function of pressure, the κ_T_ at 293.15 K is found to be 9.22 × 10^–5^ bar^–1^.^57^ The κ_T_ of the MP2-based AFM model is 9.2 × 10^–5^ bar^–1^ when estimated by finite difference of density values at −49 and 51 bar.

The surface tensions, γ, of the liquid forms of the alkanes are reported in Table 3. The simulations were performed using a liquid slab that is at least 4.3 nm × 4.3 nm in the *x*–*y* dimension and at least 4.3 nm thick in *z* dimension. An additional 10 nm vacuum is added to separate the two interfaces of the slab. A 2 nm van der Waals cutoff is used to minimize error introduced by the truncation of long-range dispersion interactions. A good agreement can be seen between the AFM prediction and experiments. Experiments show that γ of cycloalkanes is substantially larger than that of the corresponding linear alkanes. For example, cyclopentane has a γ that is 6 mN/m higher than *n*-pentane. For the linear alkanes, the γ increases as a function of chain length. However, the increase does not seem to be linear. While *n*-pentane shows a small increase of less than 1 mN/m when compared to *n*-butane, the difference between *n*-pentane and *n*-octane is almost 6 mN/m, thus the per carbon difference is much higher. The MP2-based AFM model clearly captures all these trends, although the absolute values are slightly underestimated. Truncating the van der Waals interaction at a finite value of 2.0 nm is at least partly responsible for the underestimation.^49^

**Table 3:** Surface Tension of the MP2-Based AFM Models and the Corresponding Experimental Values^70^

		γ (mN/m)	
substance	*T* (K)	exptl	MP2/AFM
cyclopentane	293	22.42	21.61
cycloheptane	293	27.93	26.08
*n*-butane	272	15.37	12.43
*n*-pentane	293	16	13.67
*n*-octane	293	21.71	19.69

## Conclusions and Discussion

By developing models that capture the MP2 potential energy surface with point-charge-based energy expressions using AFM, it is shown that many macroscopic properties of linear and cyclic alkanes can be predicted with a high accuracy. AFM fits these models with the sole purpose of reproducing the underlying MP2 potential energy surface. Thus, none of the parameters were tweaked to match the experiments. The dispersion parameters are determined by fitting to the SAPT E^2^ dispersion, which is more accurate than the dispersion in MP2. Considering that dispersion is expected to be crucial for such weakly bound molecules, these simulations do not imply that brute-force MD using MP2 would provide a similar level of accuracy. However, the description of electrostatic, repulsion, and bonded interactions at the MP2 level seems to be sufficiently accurate.

The CMD method described in this work allows partial charges to be determined in AFM by the eigen-decomposition of the charge product matrix. The method produced reliable charges that resulted in high-quality alkane models, although electrostatics is expected to play only a minor role for these nonpolar molecules.

One key difference between AFM-based models and traditional force fields is the absence of combining rules between unlike atoms. Traditional force fields rely almost exclusively on Lennard-Jones potentials^7,10,12^ with very few exceptions.^72−74^ The σ and ε parameters of like-atoms are used to derive the σ and ε parameters of unlike-atoms. While combining rules reduce the number of parameters, they introduce small errors in equilibrium interatomic distances. All fundamental interactions depend on the interatomic distances. Some interactions, such as the Coulombic and dispersion interactions, have a strong dependence on distances at short range. Thus, minor inaccuracies in interatomic distances could lead to reduced accuracy in all fundamental interactions. AFM, being able to fit a large number of parameters, allows the fitting of pair-specific short-range interactions, thus greatly improving accuracy.

Since the goal of AFM is to map the electronic structure potential energy surface for each molecule, no force field for the family of alkanes is developed. However, that would be an interesting future direction in the application of AFM. Another future direction is to incorporate 3-body dispersion to confirm whether it is the reason for the small systematic error in the prediction of density.

## Supporting Information Available

Detailed procedure for creating the hydrocarbon models using AFM, all the force field parameters for normal and cyclic alkanes, simulation details for property calculations and derivation of long-range correction to energy and pressure as a result of the *C*_8_/*r*^8^ term (PDF)
