# Supplementary material for: Simulation of Linear and Cyclic Alkanes with Second-Order Møller–Plesset Perturbation Theory through Adaptive Force Matching
Source: J Chem Theory Comput. 2024 Jun 7;20(12):5241–9. doi: 10.1021/acs.jctc.4c00509 (PMC11209940; doi:10.1021/acs.jctc.4c00509)
Supplement: Supplementary file 1 — ct4c00509_si_001.pdf [file ct4c00509_si_001.pdf]

Supporting Information

Simulation of linear and cyclic alkanes with 2nd order Møller Plesset perturbation theory through adaptive force matching

Alexei Nikitin and Feng Wang*

Department of Chemistry and Biochemistry
University of Arkansas,
Fayetteville, AR 72701, USA

* fengwang@uark.edu

1. Procedure for creating the hydrocarbon models using AFM.

Initial guess force field and conformation

A set of conformations sampled with an initial guess force field is needed to start the AFM iterations. For this purpose, the Abalone program developed by Alexey Nikitin is used. The initial guess force fields were generated using the X75 program that has been tested to work with a range of hydrocarbons. Note that this program and the autogenerated model is only used to generate the conformations for the first generation of AFM. After the first generation, sampling will be done with Gromacs using the models developed by AFM.

Sampling step of AFM

The sampling step of AFM is simulated with a cubic box containing 125 molecules using either the initial guess model or generated AFM force fields. For most alkanes, two simulations were performed at 298 K and 328 K with a 0.5 fs and for 5 ns at each temperature. For butane, the sampling was performed at the normal boiling temperature of 270 K and 328 K. For each molecule, the lower temperature sampling is performed under NPT condition using the stochastic rescaling thermostat and Berendsen barostat. The 328 K simulations were performed under NVT condition to avoid accidental vaporization for some lower boiling temperature molecules. The higher temperature simulation helps sampling the short-range repulsive region of the model.
200 snapshots are saved for each temperature from the last 4 ns of trajectory with one conformation every 20 ps. Thus for each generation 400 conformations are used for the QM calculations.

QM step of AFM

As mentioned in the paper, no MM region is used when computing reference forces for AFM as all the alkanes being studied are non-polar molecules. Neglecting MM particles will eliminate the QM/MM boundary and allow more QM particles to be fit. The QM cluster will be created by the following two step procedure.
1. One random molecule is selected to be the first molecule of the QM region.
2. Randomly select up to 5 molecules that are within 2.6 Å from the first molecule measured using nearest atom distances.
This procedure will produce a QM cluster with up to 6 molecules.
For each QM cluster, the forces were calculated using RI-MP2 with RIJCOSX and the def2-TZVP basis set using the ORCA program.

FM step of AFM

The dispersion parameters are not optimized during AFM. As described in the paper, the dispersion parameters were fit before AFM to SAPT dimer calculations.

The atom typing and energy expressions of the final force field is described in the paper. The fits were performed using a two-step procedure with the CReate Your Own Force Field (CRYOFF) program. The first step fits molecular forces and toques only and determines intermolecular parameters. This includes partial charges and parameters for short-range repulsion interactions. The reason for fitting intermolecular forces in a separate step is to avoid strong coupling with intramolecular forces, which tends to be much larger than intermolecular forces for weakly interacting compounds. The second step is intramolecular fit, where the non-bonded interactions are fixed to the values obtained in the first step and bonded parameters are determined by minimizing

the errors in atomic forces. We also emphasize that with the CMD methods, the intermolecular fit solves the linear least square problem twice as described in the paper.

Global fit

Six iterations of AFM are performed for each molecule, with the last 4 generations fit together in the global fit to obtain the final force field parameters.

Force Field parameters

Dispersion

Table S1. The parameters for the short-range damped dispersion used for all alkanes in this work.

Types		Cx(kcal/mol·$Å^x$)	x	R_0(Å)
C3	C3	-1344.3405	6	1.932
C3	C3	-22348.680	8	1.932
C3	C2	-903.12528	6	1.932
C3	C2	-26296.969	8	1.932
C2	C2	-920.03259	6	1.932
C2	C2	-13385.065	8	1.932

Cyclopentane

Table S2. The parameters for Cyclopentane

Type	Partial-charges(e)
C2	-0.23188
HC	0.11594

Types		Repulsion	Å(kcal/mol)	α($Å^{-1}$)
C2	C2	EXP	416646.82	3.8617597
C2	HC	EXP	9026.0357	3.9590755
HC	HC	EXP	814.17572	3.4747912

Bond $U = K_r/2 * (R - R_{eq})^2$

Types		R_{eq}(Å)	K_r(kcal/mol)
C2	C2	1.5421551	470.79362
C2	HC	1.0924273	733.96341

Angle $U = K_\theta/2 * (\theta - \theta_{eq})^2$

Types			θ_{eq}(degrees)	K_θ(kcal/mol)
C2	C2	C2	112.16193	152.93157
HC	C2	C2	104.52906	91.415383
HC	C2	HC	100.00734	72.890812

Torsion $U = A_t * [1 + \cos(m * \varphi - \varphi_0)]$

Types				A_t(kcal/mol)	m	φ_0(degrees)
C2	C2	C2	C2	1.1055377	3	0

Cycloheptane

Table S3. The parameters for Cycloheptane

Type	Partial-charges(e)
C2	-0.27654
HC	0.13827

Types			Repulsion Å(kcal/mol)	α(Å^{-1})
C2	C2	EXP	446937.99	3.8828860
C2	HC	EXP	10066.797	3.9318790
HC	HC	EXP	1033.1730	3.6311850

Bond $U = K_r/2 * (R - R_{eq})^2$

Types		R_{eq}(Å)	K_r(kcal/mol)
C2	C2	1.5157674	500.35619
C2	HC	1.0954678	714.86331

Angle $U = K_\theta/2 * (\theta - \theta_{eq})^2$

Types			θ_{eq}(degrees)	K_θ(kcal/mol)
C2	C2	C2	112.53653	141.19437
HC	C2	C2	107.95438	87.995479
HC	C2	HC	106.59802	72.017471

Torsion $U = A_t * [1 + \cos(m * \varphi - \varphi_0)]$

Types				A_t(kcal/mol)	m	φ_0(degrees)
C2	C2	C2	C2	-0.026450320	3	0

n-Butane

Table S4. The parameters for n-Butane

Type	Partial-charges(e)
C2	-0.23260
C3	-0.41957
HC	0.13043

Types		Repulsion	Å(kcal/mol)	α(Å^{-1})
C2	C2	EXP	250650.45	3.6866970
C2	C3	EXP	93135.619	3.2684480
C2	HC	EXP	17645.523	4.2434750
C3	C3	EXP	97445.216	3.2990230
C3	HC	EXP	7203.1754	3.6804140
HC	HC	EXP	679.27903	3.3515430

Bond $U = K_r/2 * (R - R_{eq})^2$

Types		R_{eq}(Å)	K_r(kcal/mol)
C2	C2	1.5274764	514.78810
C2	C3	1.5241406	516.29911
C2	HC	1.0927415	719.92905
C3	HC	1.0932114	736.57081

Angle $U = K_\theta/2 * (\theta - \theta_{eq})^2$

Types			θ_{eq}(degrees)	K_θ(kcal/mol)
C3	C2	C2	113.10337	136.49263
HC	C2	C2	107.53825	90.134364
C2	C3	HC	107.23358	90.788493
C3	C2	HC	107.73816	88.416036
HC	C2	HC	106.35578	72.852127
HC	C3	HC	104.36326	74.557591

Torsion $U = A_t * [1 + \cos(m * \varphi - \varphi_0)]$

Types				A_t(kcal/mol)	m	φ_0(degrees)
C3	C2	C2	C3	0.85635126	3	0

n-Pentane

Table S5. The fitted parameters for n-Pentane

Type	Partial-charges(e)
C2	0.25027
C3	0.43419
HC	-0.13493

Types		Repulsion	Å(kcal/mol)	α(Å^{-1})
C2	C2	EXP	516373.03	3.8899410
C2	C3	EXP	96312.084	3.2811820
C2	HC	EXP	29181.582	4.4173270
C3	C3	EXP	203346.32	3.5561340
C3	HC	EXP	4417.5420	3.4310600
HC	HC	EXP	703.85694	3.3837580

Bond $U = K_r/2 * (R - R_{eq})^2$

Types		R_{eq}(Å)	K_r(kcal/mol)
C2	C2	1.5254760	504.90506
C2	C3	1.5248339	513.15484
C2	HC	1.0935589	716.29210
C3	HC	1.0932825	738.34783

Angle $U = K_\theta/2 * (\theta - \theta_{eq})^2$

Types			θ_{eq}(degrees)	K_θ(kcal/mol)
C2	C2	C2	112.81352	134.52808
C3	C2	C2	113.07777	136.65772
HC	C2	C2	107.52914	89.260486
CH2	C3	HC	107.34136	90.321714
CH3	C2	HC	107.60098	89.042357
HC	C2	HC	106.36139	72.974739
HC	C3	HC	104.50086	74.092233

Torsion $U = A_t * [1 + \cos(m * \varphi - \varphi_0)]$

Types				A_t(kcal/mol)	m	φ_0(degrees)
C3	C2	C2	C2	0.75519802	3	0

n-Octane

Table S6. The fitted parameters for n-Octane

Type	Partial-charges(e)
C2	-0.27032
C3	-0.42528
HC	0.13736

Types		Repulsion	Å(kcal/mol)	α(Å^{-1})
C2	C2	EXP	781608.58	4.0341790
C2	C3	EXP	152406.69	3.4292020
C2	HC	EXP	12431.777	4.0424340
C3	C3	EXP	110436.88	3.3713460
C3	HC	EXP	5371.1327	3.5216370
HC	HC	EXP	724.66289	3.3825440

Bond $U = K_r/2 * (R - R_{eq})^2$

Types		R_{eq}(Å)	K_r(kcal/mol)
C2	C2	1.5265753	497.39566
C2	C3	1.5259849	509.03016
C2	HC	1.0946173	713.89204
C3	HC	1.0934168	734.94945

Angle $U = K_\theta/2 * (\theta - \theta_{eq})^2$

Types			θ_{eq}(degrees)	K_θ(kcal/mol)
C2	C2	C2	113.14118	133.53263
C3	C2	C2	113.32088	136.09201
HC	C2	C2	107.85690	88.415947
C2	C3	HC	107.31152	90.199454
C3	C2	HC	108.01077	88.860965
HC	C2	HC	106.70755	72.336607
HC	C3	HC	104.45610	74.253080

```
Torsion   U = At * [1 + cos (m * φ - φ0)]
===================================================
Types                At(kcal/mol)   m    φ0(degrees)
---------------------------------------------------
C2   C2   C2   C2   0.64180373     3    0
C3   C2   C2   C2   0.75296536     3    0
```

Simulation Details for Property Calculations

All property computations were performed using Gromacs package 2019.6. The liquid trajectories for the density and ΔH_{vap} were simulated for 20 ns using a 0.5 fs time step size in cubic boxes containing 125 alkane molecules. The electrostatics interactions are treated with 4th order Ewald summation with a real space accuracy of 10^{-5}. The van der Waals cutoff is chosen to be 13 Å with long range correction to energy and stress due to the C_6/r^6 term applied automatically by Gromacs. As discussed in the paper, Gromacs does not support the long range correction to pressure from the C_8/r^8 term. Thus, the long range correction to energy and pressure due to the C_8/r^8 term is done manually using the formula in the next section of the supporting information. For example, for n-heptane, the long range correction to C_8/r^8 is -11 bar with a 13 Å cutoff at the average simulation density. Thus, the barostat is set to 12 bar, which is in reality 1 bar after the -11 bar correction is accounted for.

The liquid state simulations for density and ΔH_{vap} are performed at 298 K for all the molecules except for n-butane, for which the experimental boiling temperature of 272 K is used. The temperature is maintained with the Nose-Hoover thermostat with a relaxation time of 1 ps and the pressure is maintained with Parrinello-Rahman barostat with a relaxation time of 5 ps.

For computing ΔH_{vap} the gas phase energy is computed with a single molecule in a box the same size as the liquid simulation and with cutoff coulombic. In other words, the molecule won't be able to see its images in other boxes. The box size is held as constant. The equation of motion for the single molecule is integrated with the stochastic dynamics integrator with an inverse friction constant of 2.0 ps. 100 ns trajectory is used to compute the gas phase energy for each alkane molecule. The long trajectory is important for the gas phase as there is only one molecule, obtaining good statistics is not possible unless the gas phase simulation is substantially longer than the liquid phase.

The densities at elevated pressures are also computed using 20 ns MD using the same barostat and thermostat settings as those used for 1 bar except with the higher pressure.

For each molecule, the diffusion constant D were computed also with a 500 molecule cubic box at the average density at 1 bar. The simulation temperature is the same as the temperature of the available experimental data shown in Table II. The average box size was determined with 2 ns MD NPT simulations using Nose-Hoover thermostat with a relaxation time of 1 ps and the Parrinello-Rahman barostat with a relaxation time of 5 ps. After the average box size was determined, the diffusion constant was measured by fitting the mean square displacement obtained from a 2 ns NVT simulation also with Nose-Hoover thermostat but with a longer 2 ps relaxation time.

Accurate determination of surface tension γ requires long van der Waals cutoff. Table S7 summarizes the number of alkane molecules used to create the liquid slab that is at least 42 × 42 ×

42 Å. The thickness of the box is 100 Å larger than the thickness of the slab. The γ is determined using $\gamma = \frac{L_Z}{2}\left(P_Z - \frac{P_X+P_Y}{2}\right)$ based on 10 ns of NVT simulation of the slab. The time step is 0.5 fs with Nose-Hoover thermostat with a 1 ps relaxation time.

Table S7. Number of solute molecules for the slab calculation

Molecule	Num. of Moles.
n-butane	640
n-pentane	500
n-octane	315
cyclo-pentane	500
cyclo-heptane	360

Long range correction to energy and pressure due to the C_8/r^8 term.

For a pairwise potential U(r), the long range correction to dispersion can be shown to have the form,

$$U_{corr} = n_p \cdot \frac{4\pi}{V}\int_{r_{vdw}}^{\infty} U(r)\cdot r^2 dr\ , \qquad (1)$$

where n_p is the number of pairs, which is $N_A \cdot N_B$ if A and B are different atom types, and N_A and N_B are the number of particles of types A and B, respectively. It becomes $\frac{N_A \cdot (N_A - 1)}{2}$ for pairs between one type of atoms.

The correction to the system Virial is

$$W_{corr} = n_p \cdot \frac{4\pi}{V}\int_{r_{vdw}}^{\infty} \frac{\partial U(r)}{\partial r}\cdot r^3 dr\ . \qquad (2)$$

The correction to the pressure is derived from the correction to the Virial as

$$P_{corr} = \frac{W_{corr}}{3V}\ . \qquad (3)$$

When truncating a $-\frac{C_6}{r^6}$ term, it can be shown that the long-range correction to the energy and pressure based on the above formula are

$$U_{corr} = -\frac{4\pi}{3}\cdot n_p \frac{C_6}{r_{vdw}^3},$$

(4)

and

$$P_{corr} = -\frac{8\pi}{3V^2} \cdot n_p \frac{C_6}{r_{vdw}^3} \quad (5)$$

respectively.

The long range correction to the energy (Eq. 4) is for the whole box and is not per particle. It can be shown that for one type of particles of a large number *N*. Plugging in $n_p = \frac{N^2}{2}$, we get the usual formula used by Gromacs.

$$U_{corr} = -\frac{2\pi}{3} \cdot \frac{N}{V} \cdot N \frac{C_6}{r_{vdw}^3} \quad (6)$$

and

$$P_{corr} = -\frac{4\pi}{3} \cdot \frac{N}{V} \cdot \frac{N}{V} \cdot \frac{C_6}{r_{vdw}^3}. \quad (7)$$

Gromacs actually treat all different types of atoms to be equivalent but uses an average C_6. It can be shown that it is equivalent to our derivation, where the number of pairs between identical and non-identical particles are counted explicitly.

With a $-\frac{C_8}{r^8}$ term, the above derivation gives the final expression for the correction to the energy for the whole box as

$$U_{corr} = -\frac{4\pi}{5V} \cdot n_p \cdot \frac{C_8}{r_{vdw}^5} \quad (8)$$

and the correction to the pressure as

$$P_{corr} = -\frac{32\pi}{15 \cdot V^2} \cdot n_p \cdot \frac{C_8}{r_{vdw}^5} \quad (9)$$

and the number of pairs are computed as described above.
